# Comparing the Protection Imparted by Different Fraction Extracts of Garlic (*Allium sativum* L.) against Der p–Induced Allergic Airway Inflammation in Mice

**DOI:** 10.3390/ijms20194879

**Published:** 2019-10-01

**Authors:** Chia-Chen Hsieh, Keng-Fan Liu, Pei-Chun Liu, Yaw-Tsan Ho, Wei-Sung Li, Wen-Huang Peng, Jen-Chieh Tsai

**Affiliations:** 1 Department of Medicine Division of Chest Medicine, Cheng Ching Hospital, No. 966, Sec. 4, Taiwan Road, Taichung 40764, Taiwan; pokyhsieh@gmail.com; 2 School of Chinese Pharmaceutical Sciences and Chinese Medicine Resources, China Medical University, No. 91, Hsueh-Shih Road, Taichung 40402, Taiwan; cell77821@yahoo.com.tw (K.-F.L.); weisungli@tari.gov.tw (W.-S.L.); 3 Department of Medicinal Botanicals and Health Applications, Da-Yeh University, No. 168, University Road, Chunghua 30012, Taiwan; ivy86825@gmail.com; 4 Department of Emergency Medicine, Taipei Tzu Chi Hospital, Buddhist Tzu Chi Medical Foundation, No. 289, Jianguo Road., Xindian District, New Taipei City 23142, Taiwan; yth471105@tzuchi.com.tw; 5 Plant Pathology Division, Taiwan Agricultural Research Institute, Council of Agriculture, Executive Yuan, Wufeng 41362, Taiwan

**Keywords:** garlic, asthma, Der p, anti-inflammatory, Th1/Th2

## Abstract

Garlic (*Allium sativum* L.) has been used extensively as a food ingredient and medicinally, but the effect on asthmatic airway inflammation has not been studied in detail. We accordingly explored the protective effects exerted by various garlic fraction extracts against airway inflammation with *Dermatophagoides pteronyssinus* (Der p)–induced allergic asthma in vivo and in vitro. Garlic extraction was realized using n-hexane, dichloromethane, ethylacetate, n-butanol, and water in sequence to obtain different fraction extracts. Mice were orally administered different fractions (80 mg/kg) daily for four weeks. The histological results showed that the water fraction could ameliorate lung-based goblet cell hyperplasia, inflammatory cell infiltration, and mucus hypersecretion. The water fraction extracts decreased IgE and IgG1, and they decreased inflammatory cells as quantified in bronchoalveolar lavage fluid (BALF); however, they increased IgG2a in serum. Moreover, the water fraction extracts increased IFN-γ and IL-12 (both constituting Th1 cytokines) in BALF, but they reduced IL-13, -4, and -5 (all constituting Th2 cytokines), and also inhibited the expression of IL-1β, IL-6, and TNF-α. The water fraction also inhibited the PI3K/Akt/NF-κB signal pathways in A549 cells. These findings suggest that water fraction extracts of garlic have a clear anti-inflammatory effect on Der p–induced allergic asthma.

## 1. Introduction

Characterized by sensitive trachea and bronchial inflammation, asthma currently constitutes a frequently occurring chronic inflammatory respiratory disease [1]. Asthma usually involves different reversible airflow obstruction, resulting in repeated wheezing, shortness of breath, and rapid coughing that occurs most often in the morning and at night [2,3]. Environmental and climate changes have engendered a rise in the incidence of asthma worldwide. Incidence rates for allergic asthma are particularly high in children, leading to the condition taking on particular importance [4].

Asthma symptoms are triggered by a variety of immune, genetic, and environment-related interactions [5,6]. The activation of Type 2 T helper (Th2) cells is associated with inflammatory reactions, and this causes tracheal hypersensitivity owing to cytokine release, including interleukin (IL)-13, -5, and -4 [7,8]. Additionally, B cells are spurred by Th2 cells to activate mast cells, which release more inflammatory and allergic mediators, leading to Immunoglobulin E (IgE) production [9]. Corticosteroids are the most commonly used drugs against asthma clinically [10]. However, these drugs are unable to prevent asthma symptoms in some patients. Furthermore, these drugs can produce unacceptable side effects, which limit their application. Therefore, new drugs must be developed that directly alleviate the symptoms of asthma without excessive side effects.

Previous studies indicated that administration of *Dermatophagoides pteronyssinus* (Der p) significantly increased pro-inflammatory cytokines such as interleukin-6 (IL-6), interleukin-1β (IL-1β), and tumor necrosis factor-α (TNF-α) in mice lung tissues [11]. IL-6 play a crucial role in the production of Th2 cytokines and inflammatory cell recruitment in the asthmatic airways of mice [12]. Increased inflammatory cytokines in the airway epithelium, such as IL-1β, IL-4, and TNF-α, were also observed in asthma [13]. Phosphoinositide 3-kinases (PI3K)/Akt and nuclear factor-κB (NF-κB) pathway participate in the regulation of inflammatory mediators [14]. NF-κB is a key transcription factor in the pathogenesis of asthma, and phosphorylation of NF-κB regulates the physiology of many cells, including inflammation, immune response, apoptosis, and cell death [15,16].

Garlic (*Allium sativum* L.) of the family Liliaceae is a perennial herb with a well-known and pungent odor. Garlic has been widely used as a food ingredient and spice since ancient times. Garlic also has favorable physiological activity, and it has been used to treat various conditions such as cardiovascular disease [17] and hyperlipidemia [18]. The major active components of garlic are sulfur compounds containing alliin, allicin, diallylsuifide, diallyl disulfide, diallyltrisulfide, and S-allylcysteine (SAC) [19]. A previous study found that water extracts of garlic can increase T cell cytotoxicity and lymphoproliferative capacity [20]. In Taiwan, garlic is used as a traditional folk medicine to prevent cold, cough, asthma, and bronchitis. Some people also place garlic in boiling water to obtain garlic water and drink it to prevent asthma attacks. In our previous studies, we have confirmed the protective effect of purified garlic extract on dust Der p-induced allergic airway inflammation in mice [21]; however, the mechanisms and the active components are unclear. To investigate, we used different polar solvents to obtain stratified extracts of garlic and estimated their effects on and relevant mechanisms behind mouse allergic airway inflammation engendered by Der p.

## 2. Results

### 2.1. Effects Exerted on Total Inflammatory Cell Count in Bronchoalveolar Lavage Fluid (BALF)

**Figure 1:**
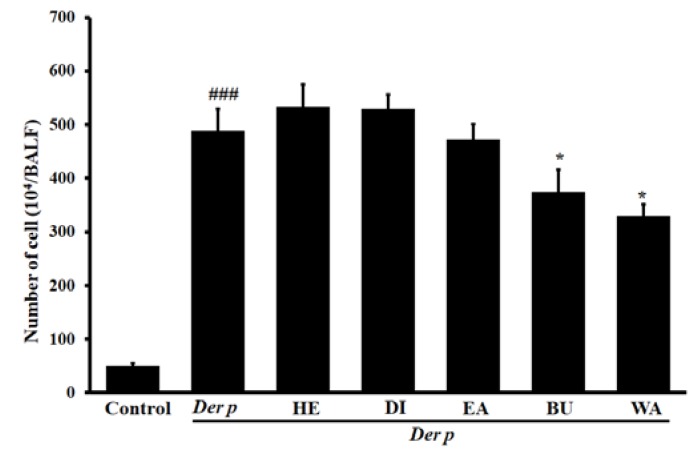
Effects of different fractions of garlic extracts (80 mg/kg) on the total inflammatory cell count in the collected bronchoalveolar lavage fluid (BALF) of mice with Der p–induced allergic asthma. Data derived are presented herein as mean ± SEM. ^###^ *p* < 0.001 versus the control group, * *p* < 0.05 versus the Der p group.

Relative to the control group, the total inflammatory cell count determined in the asthmatic mice was significantly increased, as seen in Figure 1 (*p* < 0.001). However, the mentioned count was significantly reduced by treatment with water fraction extracts (WA) and n-butanol fraction extracts (BU) at 80 mg/kg (*p* < 0.05). Moreover, as revealed by the results, no significant difference was revealed in the treatment with n-hexane fraction extracts (HE), dichloromethane fraction extracts (DI), and ethyl acetate fraction extracts (EA).

### 2.2. Pathological Results on Der p–Induced Lung and Trachea Injuries

**Figure 2:**
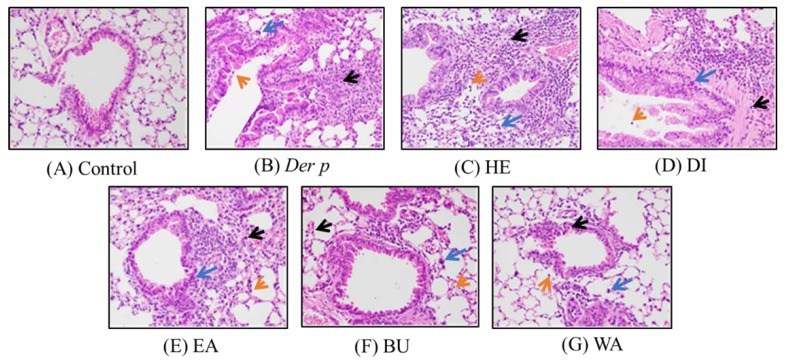
Histopathological findings in the lungs of mice with Der p–induced allergic asthma. Eosinophil is indicated by black arrows, macrophage is indicated by blue arrows and lymphocyte is indicated by orange arrows. (**A**) control group; (**B**) Der p group; (**C**) n-hexane fraction group; (**D**) dichloromethane fraction group; (**E**) ethyl acetate fraction group; (**F**) n-butanol fraction group; (**G**) water fraction group. (H&E staining, original magnification 400×).

Histopathology results revealed that, when exposed to Der p, the lung tissues of the acquired mice expressed moderate inflammatory cell infiltration, mainly eosinophils and lymphocytes, around perivascular and per bronchial space when compared with the control group, as seen in Figure 2A,B. The morphology of the eosinophils and lymphocytes in Figure 2C are as follows: Eosinophils have a bi-lobed nucleus, with highly condensed chromatin, express numerous granules stain various shades of orange, pink, or red with eosin in the cytoplasm. Lymphocytes have a relatively large, dense, often eccentric nucleus that is round and may be slightly indented. The histological changes are evaluated following previous study [22], and the results are presented in Table 1. In comparison with the Der p control group, WA fraction exhibited decreased inflammatory cell infiltration in the lungs, as seen in Figure 2G; however, inflammatory cell infiltration in the HE, DI, EA, BU fractions was not significantly reduced, as seen in Figure 2C–F, respectively. These results indicated that, in asthmatic mice, the WA fraction can inhibit Der p–induced inflammatory infiltration.

**Figure 3:**
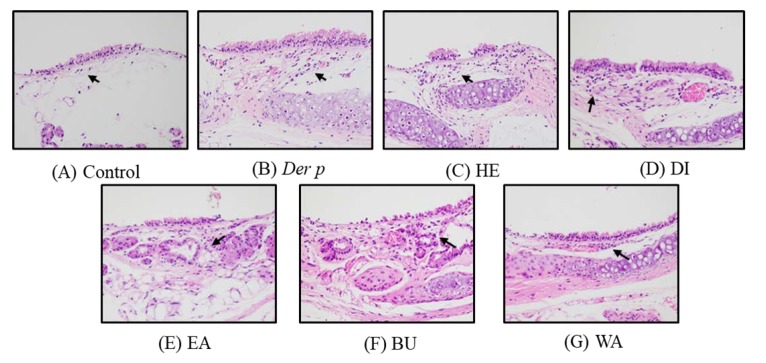
Histopathological findings in the bronchus of mice with Der p–induced allergic asthma. Inflammatory cell infiltration is indicated by black arrows. (**A**) control group; (**B**) Der p group; (**C**) n-hexane fraction group; (**D**) dichloromethane fraction group; (**E**) ethyl acetate fraction group; (**F**) n-butanol fraction group; (**G**) water fraction group. (H&E staining, original magnification 400×).

Moreover, bronchial epithelial hyperplasia and goblet cell modification in the bronchus were observed alongside macrophage aggregation in the terminal bronchioles, as seen in Figure 3A,B. The histological changes are evaluated following previous study and the results are also presented in Table 1. In comparison with the Der p control group, the WA fraction group exhibited decreased infiltration in the bronchus, as seen in Figure 3G; however, monocyte infiltration was not significantly reduced in the HE, DI, EA, and BU fractions, as seen in Figure 3C–F. Thus, in asthmatic mice, the WA fraction seemingly inhibits Der p–induced inflammatory infiltration.

### 2.3. Effects on Der p–specific serum levels of Immunoglobulin G2a (IgG2a), Immunoglobulin G1 ( IgG1) and IgE

**Figure 4:**
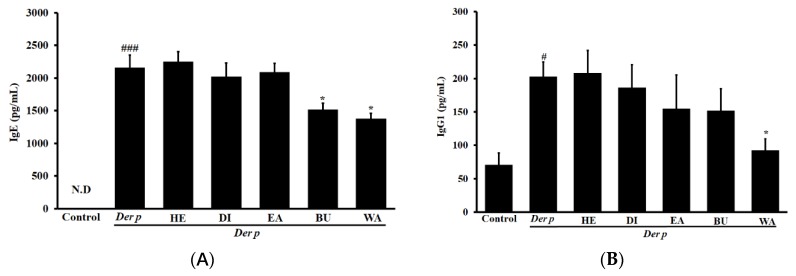

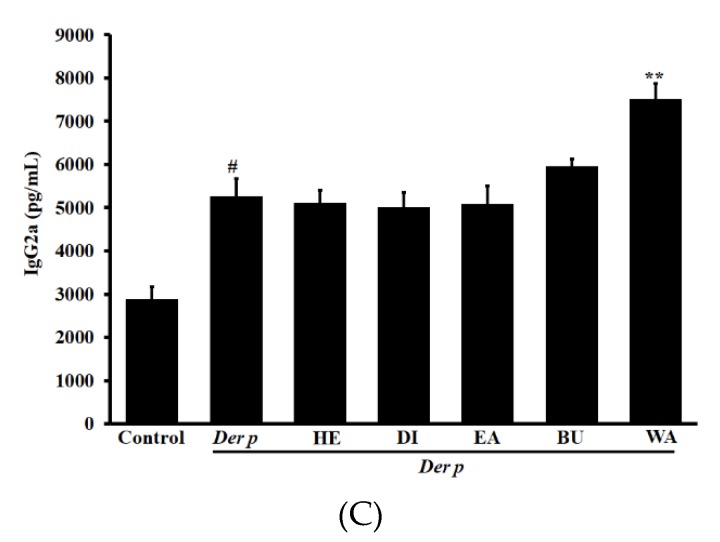
Effects of different fractions of garlic extracts (80 mg/kg) on serum (**A**) IgE, (**B**) IgG1 and (**C**) IgG2a in mice with Der p–induced allergic asthma. Data are expressed as mean ± SEM. ^#^ *p* < 0.05 and ^###^ *p* < 0.001 versus the control group, * *p* < 0.05 and ** *p* < 0.01 versus the Der p group.

Significant elevations were noted in the serum levels of IgE and IgG1 in the mice with Der p–induced allergic asthma relative to the included controls (*p* < 0.05), as outlined in Figure 4A,B. However, 80 mg/kg of the WA and BU was determined to significantly attenuate the observed serum levels of IgE (*p* < 0.05), and 80 mg/kg of the WA was noted to significantly attenuate the observed serum levels of IgG1. Conversely, the WA (80 mg/kg) also increased IgG2a serum levels, as seen in Figure 4C (*p* < 0.01). No significant differences were noted following treatment with the HE, DI, and EA.

### 2.4. Effects on Cytokine Levels in the BALF of Mice with Der p–Induced Allergic Asthma

**Figure 5:**
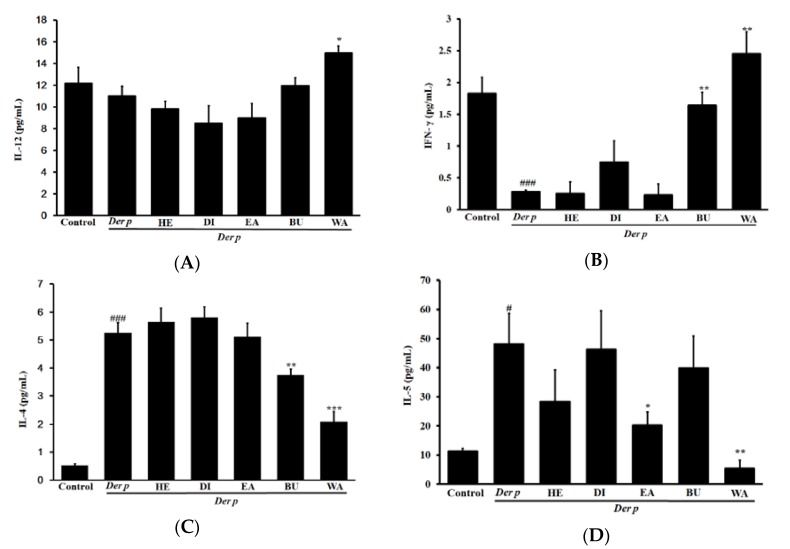

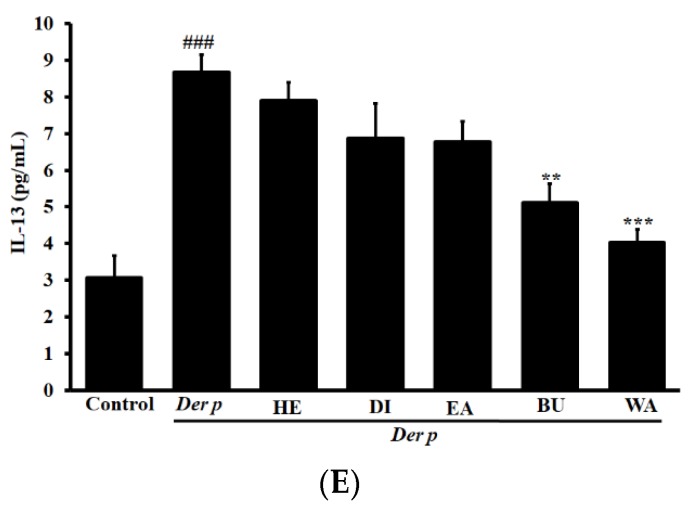
Effects of different fractions of garlic extracts (80 mg/kg) on (**A**) IL-12, (**B**) INF-γ, (**C**) IL-4, (**D**) IL-5 and (**E**) IL-13 cytokine levels in the collected BALF of mice with Der p–induced allergic asthma. Data derived are presented herein as mean ± SEM. ^###^ *p* < 0.001 versus the control group, * *p* < 0.05, ** *p* < 0.01 and ***p < 0.001 versus the Der p group.

A Th1 and Th2 cytokine imbalance is characteristic of bronchial and tracheal inflammation in asthma. Therefore, to determine the extent to which different fractions of garlic extracts modulate this imbalance, this study quantified the Th1 and Th2 cytokine levels. As displayed in Figure 5, in the Der p group’s BALF, the Th2 cytokine levels of Interleukin (IL)-13, -4, and -5 were noted to increase significantly, but the Th1 cytokine levels of Interleukin-12 (Il-12) and INF-γ were noted to decrease significantly. After treatment executed using different fractions of garlic extracts, the WA fraction decreased the levels of the observed Th2 cytokines IL-13, -4, and -5 but increased the levels of the Th1 cytokines IL-12 and INF-γ when compared with the Der p group. However, the HE, DI, EA, and BU fractions exhibited neither reductions in the observed levels of the Th2 cytokines nor increases in the observed levels of the Th1 cytokine.

### 2.5. Effects on Pro-Inflammatory Cytokine Levels in Der p–Induced Lung Tissues of Mice

**Figure 6:**
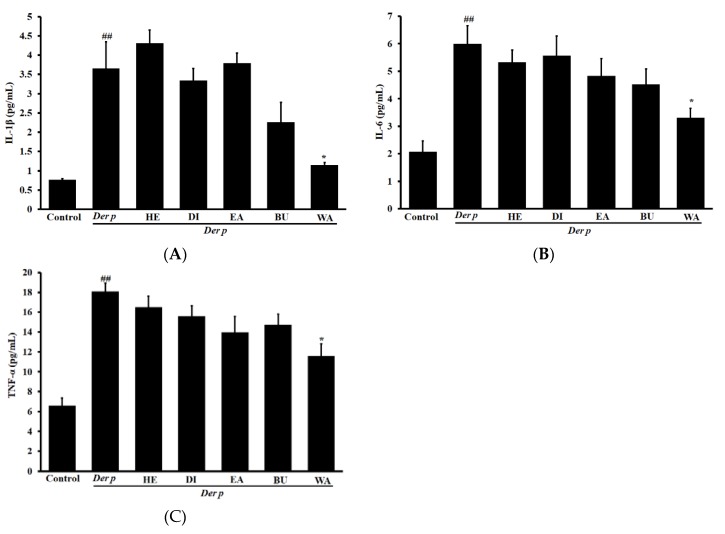
Effects of different fractions of garlic extracts (80 mg/kg) on (**A**) IL-1β, (**B**) IL-6 and (**C**) TNF-α pro-inflammatory cytokine levels in the collected lung tissue of mice with Der p–induced allergic asthma. Data derived are presented herein as mean ± SEM. ^##^ *p* < 0.01 versus the control group, * *p* < 0.05 versus the Der p group.

Figure 6 displayed the results of activations of IL-1β, IL-6, and TNF-α in the lungs of various fraction extract groups and Der p group. The levels of IL-1β, IL-6, and TNF-α in the Der p group were higher than control group. However, treatment the with WA significantly reduced the levels of IL-1β, IL-6, and TNF-α. However, there were no significant changes by treatment with the HE, DI, EA, or BU fractions.

### 2.6. Effect of Water Fraction Extracts (WA) on IL-6/PI3K/Akt/NF-κB Pathway in Der p-Stimulated A549 Cells

**Figure 7:**
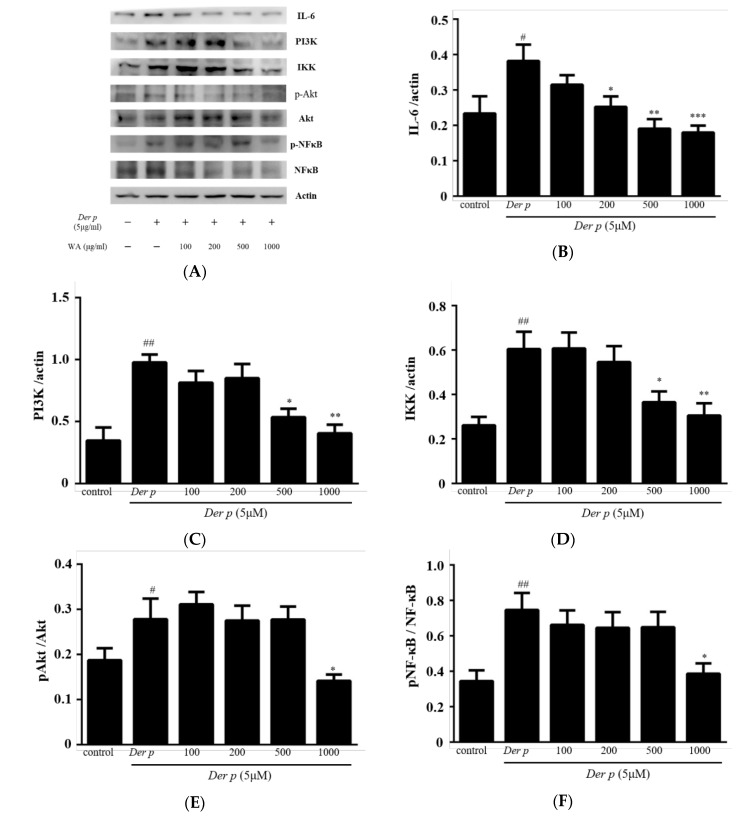
Effect of WA fraction on IL-6/PI3K/Akt/NF-κB expression in Der p-stimulated A549 cells. The cells were pretreated without (−) treatment (Control), with (+) Der-p alone or Der-p plus various concentrations (100, 200, 500, and 1000 μM) of WA fraction for 24 h (**A**). Proteins isolated from A549 cells were probed with antibodies against (**B**) IL-6, (**C**) PI3K, (**D**) IKK, (**E**) p-Akt, and (**F**) p-NFκB. The same membrane was re-probed with the antibody for β-actin to verify that equal amounts of protein were loaded. Data derived are presented herein as mean ± SEM. ^#^ *p* < 0.05 and ^##^ *p* < 0.01 versus the control group, * *p* < 0.05, ** *p* < 0.01 and *** *p* < 0.001 versus the Der p group.

In the present study, we evaluated whether WA fraction inhibited the IL-6/PI3K/Akt/NF-κB pathway in in Der p-stimulated A549 cells by Western blotting methods. As shown in Figure 7, it was observed that WA fraction significantly suppressed the gene expression of IL-6, PI3K, and IκB kinase (IKK). Additionally, WA fraction also inhibited and phosphorylations of Akt and NF-kB compared with Der p group. Thus, these results indicated that WA fraction inhibited the IL-6/PI3K/Akt/NF-κB pathway in Der p-stimulated A549 cells.

### 2.7. High-Performance Liquid Chromatography (HPLC) Analysis of the WA Fraction

**Figure 8:**
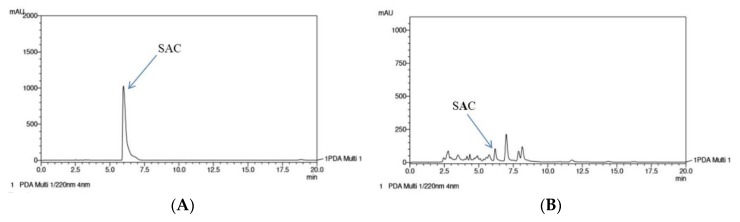
High-performance liquid chromatography (HPLC) chromatographs of the (**A**) standard and (**B**) water fraction of garlic extract. SAC: S-allylcysteine.

As shown in Figure 8, the peak of SAC was detected at a retention time of 5.97 min in the standard chromatogram. A similar peak was revealed in the WA chromatogram, suggesting the presence of SAC in the water fraction extract of garlic.

## 3. Discussion

Asthma is marked by high concentrations of Th2 cytokines and increased immunoglobulin IgE and IgG1 [23,24]. A Der p–induced animal model is considered a suitable platform for studying allergic asthma because it shares many symptoms with human asthma [25]. This model is characterized by a thickening of the bronchial wall, airway mucosal edema, excessive mucus secretion, and increased inflammatory cell infiltration into the lung [26]. By using mice with Der p–induced allergic asthma, we executed the current study to evaluate the effects exerted by different fractions of garlic extracts. In our previous study, garlic extracts inhibited Der p-induced asthmatic airway inflammation through regulation of IgE and IgG1a levels and restoring the balance of Th1 and Th2 cytokines [21]. In the current study, we evaluated the effects and the mechanisms exerted by different fraction extracts of garlic using Der p–induced allergic asthma in mice.

Our results reveal that the WA fraction significantly reduced the observed serum levels of IgE and IgG1, inhibited the creation of chemokines, increased the levels of serum IgG2a, and balanced Th1 and Th2 cytokines in mice with Der p–induced allergic asthma. This is the first time that a WA fraction of garlic extract has been shown to grant protection against allergic asthma engendered by Der p in a murine model. The primary virulence factor of asthma is the airway, and it is related to an imbalance of Th1 and Th2 cell hormones alongside increases in various immune cells and inflammatory mediators [27].

The extent of inflammatory cell infiltration in the lung affects asthma severity. Accumulation of eosinophils and lymphocytes around perivascular and per bronchial spaces causes bronchial epithelial hyperplasia and goblet cell modification in the bronchus, in addition to causing macrophage aggregation in the terminal bronchioles and alveolar spaces [28]. In the present study, Der p–induced asthmatic mice exhibited an elevation in the total cell count in the BALF. Histopathological analysis of their lung tissue revealed an increase in inflammatory cell infiltration in the lungs. However, treatment with the WA effectively inhibited the total cells, reduced inflammatory cells, and decreased inflammatory cell infiltration. Together, these findings point toward treatment with the WA having an anti-inflammatory effect on Der p–induced allergic asthma.

The primary role of chemokines is the migration of chemotactic cells, which migrate to the source of the chemokines according to the signal given by increased chemokine concentration [29]. Some chemokines control immune cell chemotaxis. Through releasing cytokines, T lymphocytes execute an integral function in immune and inflammatory response regulation processes [30]. In respiratory diseases, the literature confirms imbalance and dysfunction of Th1 and Th2 cells in the subpopulation of helper T lymphocytes as a key mechanism leading to allergic asthma [31]. Respiratory tract inflammation in asthma can be caused by Th2 cytokines [32,33]. In the immune system, Th2 cytokines, namely IL-5, -13, and -4, regulate the creation of IgE [34]. IL-13, -4, and -5 are expressed abnormally in the allergic asthma mouse model [8]. In the present study, Th2 as well as Th1 cytokines were examined in mouse BALF. The results reveal the Der p–induced mouse Th2 cytokines IL-13, -4, and -5 to be significantly increased and IFN-γ and IL-12 (both constituting Th1 cytokines) to be significantly reduced. The WA fraction of garlic extract was noted to decrease the IL-13, -4, and -5 levels but to further increase IFN-γ levels. These findings are consistent with those from cell number analysis conducted on mouse BALF and with those from pathology analysis of mouse lung tissue. Overall, these results indicate that a WA of garlic lessens airway inflammation by regulating the Th1 and Th2 cytokine balance (i.e., Th2 cytokines are reduced and Th1 cytokines are increased).

The serum levels of Der p-specific immunoglobulins IgE and IgG1a were significantly reduced after treatment with the WA fraction. Previous studies have found that IL-13 and -4 stimulate B cells to synthesize IgE, a process associated with mast cell degranulation by cross-linking with IgE receptors [30,31]. Transcription factor NF-κB activation is affiliated with the Th2 cytokine creation as well as airway inflammation during allergic reactions [35]. Conversely, NF-κB signaling pathway inhibition was found to reduce Th2 cytokine creation and airway inflammation in asthmatic mice [36]. In this study, Der p–specific IgE inhibition engendered by WA fraction treatment might have inhibited the phosphorylation of NF-κB and mediated IL-13 and IL-4 switching to IgE.

NF-κB is a downstream component of the IL-6/PI3K/Akt pathway and is activated by phosphorylation of IKK by the PI3K/Akt pathway, resulting in IκB degradation. Thus, the Il-6/PI3K/Akt/NF-κB pathway causes enhanced inflammatory expression in Der p-induced allergic asthma. This study indicated that the PI3K/Akt/NF-κB pathway is an effective target for the WA fraction against inflammation response induced by Der p in A549 cells. The WA fraction expressed anti-inflammatory activity by inhibiting the IL-6/PI3K/Akt/NF-κB pathway.

Allergic asthma is a chronic respiratory disease. Reactive oxygen species (ROS) in the environment can cause oxidative damage to cells, and it is very likely to participate in the process of dust mites promoting sensitization. During the inflammation of the lungs, the imbalance of oxidants and antioxidants in the body is an important factor in causing cell damage. ROS is involved in maintaining the “redox homeostasis” of cells to protect cells from oxidative stress. However, ROS is produced in excess, the most common cause of which is the promotion of inflammatory cytokines such as NF-κB. Oxidative stress is a harmful process that can cause damage to the airways and lungs. A previous study demonstrated that garlic essential oil and three of the individual compounds (diallyl trisulfide, ajoene, and allicin) inhibited spontaneous ROS production by neutrophils [37]. Moreover, PI3K plays an important role in the regulation of ROS production by human neutrophils. In this study, we demonstrated that WA fraction expressed anti-inflammatory activity by inhibiting the IL-6/PI3K/Akt/NF-κB pathway. Also, the pathological changes of airway were reversed after treating with WA fraction. Thus, the inhibition of ROS production may be one of the possible mechanisms in this work.

Mechanisms of allergic inflammation can contribute to the development of atherosclerosis and the pathogenesis of its clinical manifestations. Also, allergy-related cells such as basophils and mast cells may play a role in the regulation of lipid metabolism. Furthermore, it had been reported that allergic asthma and serum total IgE are associated with formation of arterial thrombosis [38]. In this study, we demonstrated that WA fraction will lower the IgE expression. Thus, the WA fraction of garlic extract may have potential in decreasing the allergy-related cardiovascular problems.

Recently, studies have indicated that obesity may have an effect on the induction of asthma as underlying the enhancement of asthma-associated inflammation due to increased (unbalanced) production of proinflammatory mediators in overgrown, inflamed, and dysregulated fat tissue [39]. In this study, we reported that WA fraction of garlic extract is effective on the restoration of Th1/Th2-related cytokine expression. Thus, we proposed that WA fraction of garlic extract may have positive effects on the treatment of obesity-induced allergic asthma.

The results of this study reveal that the WA fraction expressed the greatest anti-inflammatory effect in mice with Der p–induced allergic asthma. These findings are of some interest and indicate that the water-soluble compounds of garlic are likely to contain major active components. A previous study indicated that sulfide-derived compounds play major roles in the pharmacological activities of garlic [40]. SAC is an important hydrophilic component of garlic, and it was observed in the HPLC profile in this study. Previous research has found that SAC has anti-inflammatory activity [41]. It is therefore worth investigating further whether SAC or other water-soluble components of garlic act against Der p–engendered allergic asthma.

## 4. Materials and Methods

### 4.1. Chemicals and Drugs

Dichloromethane, ethylacetate, n-butanol, and n-hexane were purchased from Sigma-Aldrich (Sigma-Aldrich Co., St. Louis, MO, USA). Akt, p-Akt, IL-6, NF-κB, p-NF-κB and PI3-K antibodies were purchased from Abcam, Cambridge, USA. Ikk antibody was purchased from Cell Signaling Technology Danvers, USA. 1 g of Der p (Thermo Fisher Scientific, Waltham, MA, USA) was extracted using diethyl ether and then the filtrate was lyophilised to obtain Der p) extract. The extract was dissolved in sterile saline, filtered through a 0.22 μm filter, and stored at −80 °C before use.

### 4.2. Preparation of Extracts

**Figure 9:**
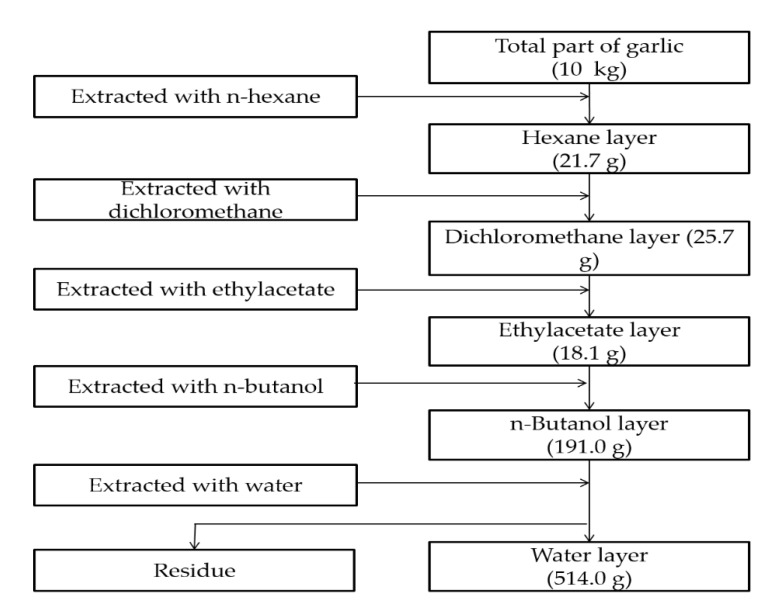
Fraction extraction procedure. The garlic was extracted using n-hexane, followed by dichloromethane, ethylacetate, n-butanol, and water.

Garlic was collected in Taichung City, Taiwan. The peeled and non-chopped garlic (10 kg) was weighed and extracted using various polar solvents. First, the garlic was extracted using n-hexane (20L) a minimum of three times. Filtrates were subsequently collected and concentrated to obtain HE. The residues were then extracted in sequence with dichloromethane, ethyl acetate, n-butanol, and water as in the aforementioned steps, resulting in five fractional extracts of garlic: HE (21.7 g, 0.22%), DI (25.7 g, 0.26%), EA (18.1 g, 0.18%), BU (191.0 g, 1.91%), and WA (514.0 g, 5.14%), as seen in Figure 9.

### 4.3. Experimental Animals

Male BALB/c mice that were determined to be aged six to eight weeks were procured from BioLASCO Taiwan (Taipei, Taiwan) and housed and cared for according to the recommendations set forth by the NIH Guide for the Care and Use of Laboratory Animals. The entirety of the animals were raised in a specific-pathogen-free lab involving a fixed temperature, namely 22 ± 1 °C, and relative humidity, namely 55% ± 5%, in addition to a 12-h light–dark cycle. In addition, water and regulation laboratory chow were available to the acquired animals ad libitum. The Institutional Animal Care and Use Committee of Da-Yeh University (Permit Number: 105024, 26 October 2016) ratified the procedures of the executed experiment.

### 4.4. Establishing an Allergic Asthma Model

Through random division, the animals were placed into seven groups (*n* = 10). Group 1 (control) and Group 2 (Der p) were orally given 0.9% saline water. Groups 3–7 were orally administered with 80 mg/kg of one of the extracts (HE, DI, EA, BU, and WA, respectively). The doses were chosen according to previous studies [21,42]. The investigator was blinded to the treatment groups.

**Figure 10:**
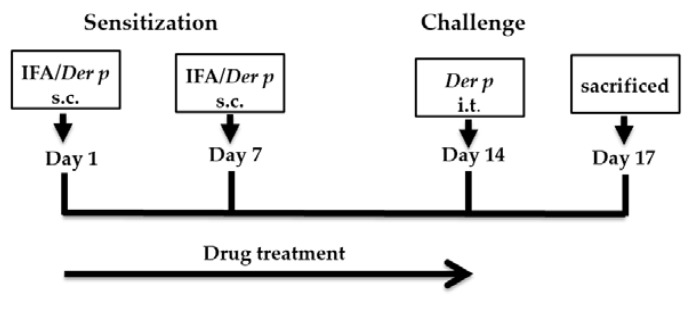
Experimental protocol of allergic sensitization and challenge.

As shown in Figure 10, on days 1 and 7, Groups 2–7 were subcutaneously injected with emulsion (50 μL) containing Der p (50 μg) in incomplete Freund’s adjuvant (Difco, Detroit, MI, USA); the injections were delivered at the base of their tails; on these days, the acquired animals in Group 1 were injected with 50 μL of saline. On day 14, a shot of tiletamine/zolazepam (*Zoletil*^®^ 50, Virbac Corporation, Carros, France), given intraperitoneally at 20 mg/kg body weight, was administered to anesthetize the mice. With the exception of Group 1, the acquired mice were subjected to treatment using an intratracheal instillation of 50 μL of Der p (1.0 mg/mL), followed by maintaining them upright for a period of 1 min until the restoration of normal breathing [43]. After 72 h since the last Der p challenge, mice were sacrificed. Lung and trachea tissues were removed for histological analysis. Serum and BALF were collected for analysis.

### 4.5. Preparing BALF and Counting Inflammatory Cells

In the executed study, total cells and BALF were collected according to He et al. [44]. Briefly, after 72 h since the last Der p induction, the mice were euthanized under anesthesia. Subsequently, the lungs were lavaged using a syringe containing 1 mL of ice sterile saline solution. This process was repeated three times, and then the lavage fluid was collected. The collected lavage fluid was subjected to a 10-min centrifugation process executed at 4 °C and 1200 rpm, followed by gathering and keeping the supernatant at −80 °C. PBS constituted the medium for resuspending each tube’s cell pellet; a hemocytometer was used to measure the total cell count.

### 4.6. Histological Lung Assessment

Each lung was placed in an embedding cassette after being fixed in 10% neutral formalin. The lung tissue was first dehydrated, and, after standing overnight, the abovementioned tissue was inserted into paraffin and chopped into approximately 4–5-μm sections by using a microtome. The experiment was performed after the paraffin was dried and fixed. After the execution of staining performed by employing hematoxylin and eosin (H&E), the specific conditions and histopathological changes of each group of lung cells were examined under a light microscope, during which the degree of injury was compared and photographed. The severity of lesions in the lungs was graded according to the methods described by Shackelford et al. [22]. The degree of lesions was graded from one to five depending on severity: 1 = minimal (<1%); 2: slight (1–25%); 3 = moderate (26–50%); 4 = moderately severe (51–75%); 5 = severe/high (76–100%). Mean histopathological scores were calculated by dividing the sum of the score per grade of affected mice by the total number of examined mice.

### 4.7. Measurement of Type 1 T helper (Th1) and Th2 Cytokine Concentrations in BALF

Following previous studies [45,46], IFN-γ and IL-12, -13, -4, and -5 levels in the lavage fluids of the lungs from the control, Der p, and garlic extract–fed mice ventilated ex vivo with either the protective or the injurious strategy were determined in the laboratory by using mice-specific ELISA kits (BioSource, Camarillo, CA, USA). For subsequent analyses, a Bio-Plex assay was executed to determine the IFN-γ and IL-12, -13, -4, and -5 levels, which were then read on a Luminex-100 system (Luminex Co., Austin, TX, USA). The analysis of the results was executed via Bio-Plex Manager software (Bio-Rad Laboratories, Hercules, CA, USA).

### 4.8. Der p–Specific Serum IgE/ IgG1/ IgG2a Concentrations

In this study, serum IgE/IgG1/IgG2a concentrations were quantified via appropriate ELISA kits per instructions provided by the relevant manufacturer (Promega, Madison, WI, USA).

### 4.9. Measurement of Pro-Inflammatory Cytokine Concentrations in Lung Tissues

In this study, the inflammatory cytokine IL-1β (ab100704) / IL-6 (ab100712) / TNF-α (ab100785) concentrations were quantified by appropriate ELISA Kits according to the instructions provided by the relevant manufacturer (Abcam, Cambridge, UK).

### 4.10. Cell Culture

A549 cells were purchased from the Culture Collection and Research Center of the Food Industry Institute (Hsin-Chiu City, Taiwan). The cells were cultured in F-12K medium containing 10% fetal bovine serum (FBS) and 1% Penicillin-streptomycin-amphotercin solution (PSA), and placed in a 37 °C, 5% CO_2_ incubator for growth. When the cells reached approximately 80% confluence, subculture was carried out; the cells were suspended in a liquid by adding 0.05% Trypsin-EDTA and cultured in a plate at a number of 1 × 10^6^ cells. After 24 hours of plating, Der p (5 μg/mL) and WA extract (100, 200, 500, or 1000 μg/mL) was added and reacted for 24 h The cells were harvested via treatment with cell lysis buffer for further studies.

### 4.11. Western Blotting Analysis

Total protein lysate from the cells was extracted in lysis buffer containing a mixture of protease and phosphatase inhibitors (Sigma-Aldrich). Protein concentration was determined using a BCA protein assay kit (Pierce Biotechnology, Rockford, IL, USA). Protein lysate (50 μg) was separated by 10% SDS-PAGE and transferred to a polyvinylidene fluoride membrane (Millipore, Billerica, MA, USA). The membrane was blocked in Tris buffered saline containing 3% bovine serum albumin in Tween at 25 °C for 1 hour. After washing, the membrane was incubated overnight at 4 °C in the following primary anti-IL-6 (1:1000), PI3-K (1:1000), *IκB kinase* (Ikk) (1:500), p-Akt (1:500), Akt (1:500), p-NF-κB (1:500), NF-κB (1:1000) antibodies. After washing the membrane with TBS-T, the blot was incubated in a 1/5000 dilution of horseradish peroxidase-conjugated secondary antibody at 25 °C for 1 hour. Protein bands were visualized using an enhanced chemiluminescence kit (PerkinElmer, Boston, MA, USA). Actin (Millipore) was used as an internal control. The optical density of the bands were determined by software.

### 4.12. HPLC Analysis of the WA

For the WA and standard (S-allylcysteine, SAC), a high-performance liquid chromatography (HPLC) profile was established. The instruments comprised a Shimadzu LC-10Avp HPLC system and SPD-M10A Diode array detector. Analyses were executed in the experiment by employing a COSMOSIL 5C18-AR II column (4.6 × 250 mm^2^, 5 µm), with the mobile phase being observed to contain acetonitrile and 10 mM KH_2_PO_4_ with an isocratic elution (2:98). All standards and samples in the experiment were passed through a 0.45-µm Minipore filter prior to injection (10 µL) into the column. A 220-nm detection wavelength was employed, and the determined flow rate was 1.0 mL/min. Each analysis required 20 min.

### 4.13. Statistical Analysis

Herein, the derived results are presented as the mean ± SEM according to each group’s sample number (n). With regard to the differences among multiple groups, the corresponding significance was evaluated using analysis of variance (ANOVA). On establishing significance between groups, Duncan’s multiple range test was employed to compare two specific groups’ mean values via SPSS for Windows, version 17 (IBM, Chicago, IL, USA). In addition, statistical significance was set in this study to be *p* < 0.05.

## 5. Conclusions

Using a Der p–engendered allergic asthma model in mice, we estimated the anti-inflammatory effects of different fractions of garlic extracts (HE, DI, EA, BU, and WA). The WA fraction had a clear protective effect against allergic asthma. Specifically, treatment with the WA fraction significantly decreased airway inflammation and reduced expression of IL-13, -4, and -5 in BALF as well as serum IgE and IgG1. Therefore, the WA fraction could regulate the imbalance between Th1 and Th2 by inhibiting Th2 cytokines and inflammatory protein. Histological results reveal that the WA fraction attenuated Der p–induced inflammatory cell infiltration in lung tissue and cell infiltration within the submucosal layer of the trachea. The WA fraction also expressed anti- inflammatory activity by inhibiting IL-6/PI3K/Akt/NF-κB signal pathways. In conclusion, the WA fraction was effective in treating Th2-type allergic responses in asthma engendered by Der p in a murine model, indicating that it might be a protective agent for use in patients with allergic asthma.

## Figures and Tables

**Table 1:** Quantitative summary of the protective effects of different fraction extracts of garlic on Der p–induced allergic asthma based on histological observations.

Organ	Histopathological Findings	Groups ^1^						
		Control	*Der p*	*Der p*				
				HE	DI	EA	BU	WH
Lung	Inflammation, eosinophilic and lymphocytic cells, perivascular and per bronchial, focal	0.0 ± 0.00	3.3 ± 0.19 ^#^	3.0 ± 0.00	2.5 ± 0.50	2.8 ± 0.19	2.8 ± 0.19	1.8 ± 0.19 *
	Aggregation, macrophage and giant cells, alveolar, focal	0.0 ± 0.00	2.5 ± 0.22 ^#^	2.0 ± 0.00	1.8 ± 0.38	1.8 ± 0.19	1.5 ± 0.22	0.8 ± 0.19 *
	Epithelial hyperplasia, bronchial, focal	0.0 ± 0.00	2.0 ± 0.00 ^#^	2.0 ± 0.00	1.5 ± 0.38	1.5 ± 0.22	1.0 ± 0.31 *	1.0 ± 0.31 *
	Mucification, goblet, bronchial, focal	0.0 ± 0.00	2.0 ± 0.00 ^#^	1.8 ± 0.19	1.5 ± 0.38	1.5 ± 0.22	1.0 ± 0.31 *	1.0 ± 0.31 *
Brachus	Inflammation, submucosal, focal	1.0 ± 0.00	3.3 ± 0.58 ^#^	2.3 ± 0.58	2.0 ± 0.82	2.0 ± 0.82	1.5 ± 0.58	1.3 ± 0.43 *

^1^ Degree of lesions was graded from one to five depending on severity: 1 = minimal (< 1%); 2 = slight (1–25%); 3 = moderate (26–50%); 4 =moderate/severe (51–75%); 5 = severe/high (76–100%). ^#^ Statistically significant difference between control and Der p groups at *p* < 0.05^.^ * Statistically significant difference between Der p and treated groups at *p* < 0.05.

## Abbreviations

Der p	Dermatophagoides pteronyssinus
BALF	bronchoalveolar lavage fluid
Th1	Type 1 T helper
Th2	Type 2 T helper
IgE	Immunoglobulin E
IgG1	Immunoglobulin G1
IgG2a	Immunoglobulin G2a
IL-12	Interleukin-12
IL-13	Interleukin-13
IL-4	Interleukin-4
IL-5	Interleukin-5
IL-6	Interleukin-6
IL-1β	Interleukin-1β
IFA	Incomplete Freund’s adjuvant
TNF-α	Tumor necrosis factor-α
PI3K	Phosphoinositide 3-kinases
NF-κB	Nuclear factor-κB
IKK	IκB kinase
HE	n-Hexane extracts
DI	Dichloromethane extracts
EA	Ethyl acetate extracts
BU	n-Butanol extracts
WA	Water extracts
SAC	S-allyl cysteine sulfoxide
